# Evaluation of the Frequency of HLA-DQ2/DQ8 Genes Among Patients with Celiac Disease and Those on a Gluten-Free Diet

**DOI:** 10.3390/foods14020298

**Published:** 2025-01-17

**Authors:** Marek K. Kowalski, Danuta Domżał-Magrowska, Ewa Małecka-Wojciesko

**Affiliations:** Department of Digestive Tract Diseases, Norbert Barlicki Memorial University Hospital, 90-153 Lodz, Poland; mekamed14@gmail.com (M.K.K.); danuta.magrowska@gmail.com (D.D.-M.)

**Keywords:** celiac disease, HLA-DQ2/DQ8 genes, gluten-free diet

## Abstract

Background: Celiac disease (CD) is a chronic, permanent, gluten-dependent disease that manifests itself with inflammation of the small intestine and malabsorption in genetically predisposed individuals with HLA-DQ2 and -DQ8 (human leukocyte antigen) histocompatibility antigens. Objective: The diagnostic criteria for celiac disease have undergone numerous modifications over the years. The aim of the study is to evaluate the frequency of HLA-DQ2/DQ8 genes in a group of patients with celiac disease diagnosed in 1980–2010 in order to verify the primary diagnosis of CD. Methods: The study group included 50 patients, 13 men and 37 women, who had been diagnosed with celiac disease many years ago based on histopathological criteria and improvement of health condition after receiving a gluten-free diet. The control group consisted of 31 healthy volunteers, 18 women and 13 men. All subjects underwent a genetic analysis assessing the presence of histocompatibility antigens HLA-DQ2.2, -DQ2.5, and -DQ8, along with the assessment of alleles encoding the α and β subunits of the antigens, according to European Society of Paediatric Gastroenterology, Hepatology and Nutrition (ESPGHAN) guidelines from 2020, using the EUROarray technique at EUROIMMUNE^®^. Results: In the study group, 12 (24%) patients did not meet the genetic criteria. Among the remaining patients (Group 1) with celiac disease, the presence of HLA-DQ2.5 (50.0% vs. 9.68%; *p* < 0.01) and the co-occurrence of both alleles of HLA-DQ2 (31.6% vs. 6.45%; *p* < 0.05) were detected significantly more frequently than in the control group. Among patients with celiac disease, the prevalence of HLA-DQ8 was also slightly more frequent (13.2% vs. 3.23%; *p* > 0.05). Patients who did not meet the genetic criteria for celiac disease (Group 2) had a single string α-HLA-DQ2.5 significantly more often than control subjects (66.67% vs. 38.71%; *p* < 0.05). Conclusions: Among patients with celiac disease diagnosed before 2010, based on the 2020 ESPGHAN criteria, it is advisable to verify the previous diagnosis, taking into account genetic criteria.

## 1. Introduction

Celiac disease (CD) is a chronic, permanent, gluten-dependent disease that manifests itself with inflammation of the small intestine and malabsorption in genetically predisposed individuals with HLA-DQ2 and -DQ8 histocompatibility antigens [1,2]. Due to the complaints and accompanying symptoms, two variants of celiac disease are distinguished with intestinal and extraintestinal manifestations [1,2].

CD is considered one of the most common autoimmune diseases. Its prevalence, depending on the studied population and methodology, is estimated at 0.75–1.6% of all screened subjects [3,4,5,6]. Among first-degree relatives of patients with celiac disease, the risk of developing the disease is 4.5–10.7% and among second-degree relatives 2.3–2.6% [7,8,9].

### 1.1. Criteria for the Diagnosis of Celiac Disease: Historical Outline and Current Status

Over the decades, the criteria for diagnosing celiac disease have gradually changed along with the increasing knowledge of its etiopathogenesis. The guidelines developed at an expert meeting in 1969 in Interlaken, Switzerland, included villous atrophy, which was reversible after a gluten-free diet and recurred following the period of gluten challenge and typical clinical symptoms (diarrhea, abdominal pain, vomiting, flatulence, and fatty stools) that disappeared after a gluten-free diet. The criteria assumed performing three diagnostic gastroscopies to obtain biopsy samples from the duodenum [10,11]. Due to the low acceptance of returning to a gluten-free diet among patients, in 1979 ESPGHAN (European Society of Paediatric Gastroenterology, Hepatology and Nutrition) allowed the diagnosis of celiac disease based on a single biopsy, provided that there was clinical improvement after the applied diet [12]. In the subsequent ESPGHAN criteria from 1990, the determination of characteristic antibodies in the diagnosis of celiac disease was recommended [13]. Only the NICE (National Institute for Health and Care Excellence) 2009 guidelines for the diagnosis of celiac disease included the determination of antibodies against tissue transglutaminase II (anti-tTG) and antibodies against endomysium (anti-EMA). The important role of HLA-DQ2/DQ8 gens in the pathogenesis of celiac disease was also recognized, however, they were not included in the criteria for the diagnosis of celiac disease [14]. Currently, in Poland the 2020 ESPGHAN guidelines for the diagnosis of celiac disease are in force, which are a modification of the 2012 criteria. According to the ESPGHAN criteria from 2012, the diagnosis included the clinical picture (both typical and atypical symptoms), villous atrophy Marsh grade 2 or 3, detection of characteristic anti-endomysial antibodies, anti-tissue transglutaminase 2 (anti-tTG) or anti-deamidated gliadin peptides (anti-DGP), at a concentration that is at least three times higher than normal, and the presence of specific histocompatibility antigens HLA-DQ2 or -DQ8. However, the 2020 modification places the main emphasis on the determination of anti-tTG IgA antibodies and the determination of the total IgA level in order to exclude a deficiency and thus a false negative result. In the case of children and adolescents, the simultaneous finding of ten times the upper limit of normal anti-tTG IgA antibodies with a normal level of total IgA and a typical clinical picture allows for the diagnosis of celiac disease omitting the necessity of histopathological examination of a duodenal biopsy [1,2].

In Poland, for years, due to low availability and the applicable criteria for diagnosing celiac disease, which did not include serological tests, specific antibody tests were not performed in accordance with the ESPGHAN guidelines from 1989 [13].

According to the above guidelines, celiac disease is very unlikely in patients who do not have HLA-DQ2 and HLA-DQ8 antigens. These human leukocyte antigens are major histocompatibility complex (MHC) class II molecules expressed on antigen-presenting cells (APCs) to present exogenous antigens and proteins (i.e., deamidated gliadin peptides) to T lymphocytes [15,16,17]. HLA class II molecules consist of two polypeptide chains (an *α* and a *β* chain), and each chain is folded into two separate domains: *α*1 and *α*2 and *β*1 and *β*2, respectively. A peptide-binding groove is formed by the distal *α*1 and *β*1 domains. The proximal domains, *α*2 and *β*2, are highly conserved to which the T cell receptor (TCR) binds [18].

### 1.2. Aim of the Study

Over the years, the guidelines for diagnosing CD have been changed as knowledge about it has deepened and new, widely available diagnostic tests have been developed, allowing for a more specific diagnosis. As a result, many patients who were diagnosed with the disease in the past do not currently meet the required criteria. The aim of the study was to verify the diagnosis of CD made in the years 1980–2010 by determining the presence of alleles encoding HLA-DQ2 and -DQ8. The frequency of the basic alleles in the general control groups was assessed. Among patients with CD who did not meet the criteria for genetic diagnosis of the disease, a similar analysis was also performed. This is the first detailed genetic analysis of patients with celiac disease in Central Europe.

## 2. Material and Methods

Initially the study included 57 patients diagnosed with celiac disease between 1980 and 2010 who were on a gluten-free diet (mean 70 months ± 90 SD). In this group, the diagnosis made many years ago was verified according to the ESPGHAN criteria from 1989. Five patients did not provide histopathological results or were unable to determine the histopathological findings of the duodenal biopsy. In 2 other patients, at the time of the diagnosis, histopathology revealed Marsh grade 1, which was insufficient to make a correct diagnosis of celiac disease in the light of the lack of other tests for celiac disease. Despite not meeting the criteria at the time of the diagnosis, these patients were on a gluten-free diet and were diagnosed with celiac disease.

After analyzing the medical history, taking into account the criterion of histopathological changes (in the study group, patients had to have villous atrophy of at least Marsh 3a) and the improvement found after a gluten-free diet, the study group included 50 patients, 13 men and 37 women. The control group consisted of 31 healthy volunteers, 18 women and 13 men.

Then, genetic analysis was performed to assess the presence of HLA-DQ2.2, -DQ2.5, and -DQ8 along with the evaluation of alleles encoding the α and β subunits of the antigens, in accordance with the applicable ESPGHAN guidelines from 2020. Evaluation of tissue antigen subunits allows the exclusion of false positive results. EUROIMMUNE^®^ EUROarray was used for the investigations. Blood samples were placed in a thermal cycler with HLA-DQA1 and HLA-DQB1 primers. Amplification products were labeled with fluorescent markers. The obtained product was applied to EUROArrays (containing BIOCHiP probes with microarrays) by the TITERPLANE technique (a technique using a hydrophilic material in the central field and a hydrophobic material outside the field, so that the samples cannot mix with each other). As a result of hybridization, a fluorescent reaction occurred at the site where the material contained HLA-DQA1 or HLA-DQB1, respectively. Microarray analysis was performed using the EUROIMMUN Microarray Scanner with EUROArrayScan software. The evaluation was performed in a fully automated device that excluded the possibility of human error and facilitated data archiving. After the analysis, in both the study group and the control group, the presence of HLA-DQA1∗05–DQB1∗02 and -DQA1∗03–DQB1∗03:02 alleles coding for the HLA-DQ2 and HLA-DQ8 antigens was confirmed or excluded. The frequency of the DQA1*0501-DQB1*0201 and DQA1*0201-DQB1*0202 alleles, which encode HLA-DQ2.5 and HLA-DQ2.2, respectively, predisposing to the development of celiac disease, was also assessed. Among patients who did not meet the genetic criteria for the diagnosis of celiac disease and in the control group, the frequency of single alleles coding the appropriate α and β chains was assessed.

### Statistical Analysis

Independent groups were compared in the analyzed study. Nominal variables are presented as percentages. To compare two nominal variables, the Chi-squared test with Yates’ correction or the two-sided Fisher’s exact test was used, depending on the size of the investigated groups.

The level of statistical significance was assumed to be *p* < 0.05.

Statistical analyses were performed using Statistica 10 (Statsoft, Tulsa, OK, USA).

## 3. Results

*Investigation of the Prevalence of HLA-DQ2 and HLA-DQ8*

**Figure 1:**
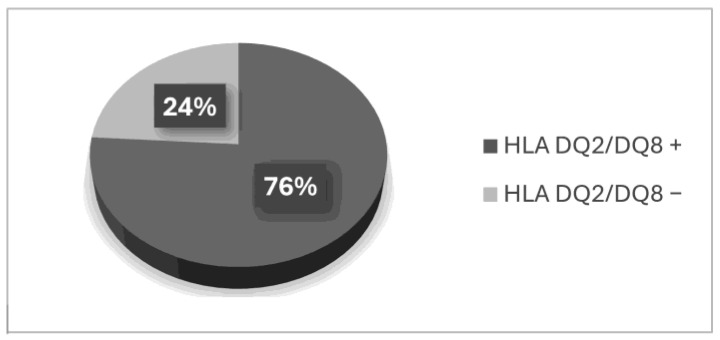
Gene frequency among patients on a gluten-free diet.

In the group of patients following a gluten-free diet who had been previously diagnosed with celiac disease, 12 (24%) did not meet the genetic criterion. Based on the performed analysis, the presence of two alleles of the HLA-DQ2 or -DQ8 antigens was not detected (Figure 1).

**Figure 2:**
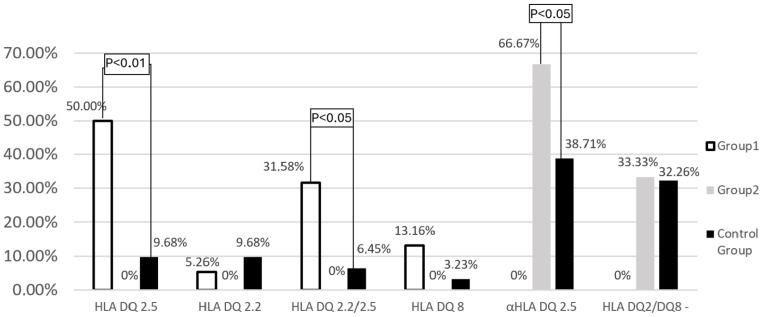
Frequency of HLA−DQ2.2, HLA−DQ2.5, and HLA−DQ8 genes in the study groups and the control group.

Among the remaining patients (Group 1) with celiac disease, the presence of HLA-DQ2.5 (50.0% vs. 9.68%; *p* < 0.01) and the co-occurrence of both alleles of HLA-DQ2 (31.6% vs. 6.45%; *p* < 0.05) were detected significantly more frequently than in the control group. Analyzing the frequency of HLA-DQ2.5 and HLA-DQ2.2 separately, the first variant was found to be significantly more frequent (81.58% vs. 36.84%; *p* < 0.01). Among patients with celiac disease, the prevalence of HLA-DQ8 was also slightly more frequent (13.2% vs. 3.23%; *p* > 0.05). Patients who did not meet the genetic criteria for celiac disease (Group 2) had a single string α- HLA-DQ2.5 significantly more often than control subjects (66.67% vs. 38.71%; *p* < 0.05) (Figure 2).

In the analyzed Group 1, flatulence was observed significantly more frequently among patients with the HLA-DQ2.5 allele than in those with other genetic variants (77.8% vs. 45%; *p* < 0.05). No other correlations were found between genetic variants and reported symptoms.

## 4. Discussion

Initially, 57 patients diagnosed with celiac disease were enrolled into the study according into the 1990 ESPGHAN criteria. These criteria, described in the Introduction, were mainly based on clinical symptoms and characteristic histopathological abnormalities in the intestinal mucosa [13]. Meanwhile, many later reports indicated the occurrence of histopathological changes, similar to those observed in celiac disease, in the course of other chronic diseases [19]. In patients with a history of celiac disease who follow a gluten-free diet, HLA-DQ2/DQ8 should be tested to verify the primary diagnosis. According to the current knowledge, the presence of HLA-DQ2/DQ8 is necessary for the development of celiac disease, and the occurrence of the disease without their presence seems very unlikely [1,2].

Genetic diagnostics to verify the previous diagnosis were used in 12 cases. In eight patients, only the HLA-DQ2 α allele was detected, whereas in four patients, no HLA-DQ2/8 was found. Despite quite unambiguous 2020 ESPGHAN guidelines, some authors drew attention to the fulfillment of other criteria for the diagnosis of celiac disease also among patients with only a single HLA-DQ2α subunit, as well as HLA-DQ7, which had not yet been considered important in the development of celiac disease and was not included in the analyzed study group. A large study by Karell et al. on a group of 1008 patients with celiac disease showed that 61 carried neither HLA-DQ2 nor HLA-DQ8 genes, and 57 of these encoded half of the DQ2 heterodimer. In the remaining four patients, none of the previously recognized genes were detected [20]. Later studies by Tinto et al. confirmed the presence of HLA-DQ2/DQ8 in only 95.8% of the study participants. In the remaining group of patients, the presence of increased frequency of HLA-DQ7 was demonstrated [21]. Similarly, in the study by Lund et al., the frequency of HLA-DQ2 and -DQ8 was assessed in 128 patients with histologically confirmed celiac disease. The presence of HLA-DQ2.5 or -DQ8 was confirmed in 120 patients. The presence of HLA-DQ2.2 or -DQ2.5 (trans) was detected in seven, and in one patient no antigens were detected [22]. Although in the study group the frequency of HLA-DQ2.5 was statistically significantly higher than HLA-DQ2.2, the difference was not so large. Turkish study involving 94 patients, the presence of predisposing genes was confirmed in 73.4% of the study participants [23]. While the rate of misdiagnosis of CD was similar to that in the study conducted above, the authors did not attempt to assess the cause of misdiagnosis and did not state when the original diagnosis was made or by what criteria. Similarly, in a meta-analysis based on data from Spain, in 3% of patients diagnosed with celiac disease many years ago, based on the clinical picture and histological changes according to the 1979 ESPGHAN criteria, the presence of HLA-DQ2 or -DQ8 was not observed. Interestingly, in the group of patients in whom the HLA-DQ2 or -DQ8 genes were not confirmed, the presence of a single chain was confirmed in 76%. This is significantly more than in the study conducted above, but less than in the earlier study by Karrell et al. The meta-analysis also draws attention to the fact that among patients with a single HLA chain, genes encoding the beta chain were observed more frequently, whereas in the above study in patients with a single HLA chain only, genes encoding the alpha chain were found, which is insufficient for antigen presentation and therefore cannot lead to the development of celiac disease [24]. In a study of 57 Iranian patients with celiac disease, no genes predisposing to the development of the disease were found in 2 patients [25]. Kapitany et al., analyzing the frequency of HLA-DQ2 and -DQ8 among 70 patients with previously diagnosed celiac disease based on histopathology, found the presence of antigens in 56% of study participants. However, in the group of HLA-negative patients, a long-term gluten challenge did not lead to an increase in the concentration of anti-tissue transglutaminase antibodies, and thus celiac disease was not confirmed in this group [26]. Similarly, Hopman et al. did not observe any increase in antibody levels during the use of gluten challenge in HLA-negative patients [27]. The studies by Kapitany et al. and Hopman et al., as well as the conducted study, clearly confirm the low efficacy of the CD diagnosis based on histopathological criteria only, since villous atrophy may occur in the course of many other diseases [19]. However, Bergsens et al., based on molecular studies, confirmed the involvement of single α chains and HLA-DQ7 in the pathogenesis of celiac disease [28]. The latest studies conducted in Italy also indicated an important role of HLA-DQ7 in the development of celiac disease [21,29]. Werkstetter et al.’s multicenter study referred to ESPGHAN, confirmed the presence of typical HLA in all 399 patients with newly diagnosed celiac disease [30]. Similarly, Wolf et al. found out that all 227 patients diagnosed with celiac disease had HLA-DQ2 or -DQ8 [31]. Further studies were conducted in France on 82, in Finland on 114, in Sweden on 153, in Pakistan on 59, and in Lebanon on 31 patients that showed the presence of typical genes in 98.8–100% of the examined patients [32,33,34,35,36]. Interestingly, Siddiqui et al. also evaluated patients with symptoms resembling celiac disease and showed the presence of a single α or β chain in 100% of cases (11 patients) [36]. Among patients initially qualified for the study who had been diagnosed with celiac disease years ago, HLA-DQ2 and -DQ8 were relatively frequently not detected, similarly to the studies cited above. In the light of the above reports and in accordance with current 2020 ESPGHAN guidelines, if patients carry a single DQ2 allele or are negative for both HLA-DQ2 and -DQ8, then it is very unlikely that they have celiac disease. At the same time, it should be noted that genetic verification of a diagnosis made years ago often questions the earlier diagnosis.

Nevertheless, the group of patients without HLA-DQ2 or -DQ8, who had celiac disease diagnosed in the past, should be recommended a gluten challenge. Rispo et al. showed that introducing 60–120 mg of gluten into the diet for 3 months is well tolerated and provides a definitive answer as to the occurrence of celiac disease [37]. Among HLA-DQ2 and -DQ8-negative patients, the use of gluten challenge did not cause any symptoms [38]. In the analyzed group, the presence of HLA-DQ2.5 was detected in 31 patients (81.58%). Previous genetic studies for celiac disease conducted among large patient populations also showed the presence of HLA-DQ2.5 in nearly 90% of patients. Similarly to the analyzed group, HLA-DQ2.2 and -DQ8 occurred much less frequently among patients with celiac disease [25,39,40,41]. It should be emphasized that detailed molecular and cytogenetic studies confirmed the presence of binding areas of gliadin peptide fragments with HLA-DQ2.2 and -DQ8 in patients with celiac disease [42,43].

In the study group, the presence of HLA-DQ2.2 was detected less frequently than in the control group (5.26% vs. 9.68%). The reason for the reduced frequency of HLA-DQ2.2 in the population of patients with celiac disease may be explained by the study by Bodd et al., who analyzed seven patients with celiac disease with confirmed presence of HLA-DQ2.2, in whom no other genes predisposing to celiac disease were detected. It was shown that in this group of patients, different areas of gliadin became immunogenic than in patients with HLA-DQ2.5 [42]. Also, Alam et al. demonstrated that the frequency of HLA-DQ2.2 among patients with celiac disease was similar (8% of the examined patients) to that obtained in Group 1 [41]. Fallang et al. showed that bindings between gliadin and HLA-DQ2-presenting cells were more stable in HLA-DQ2.5(+) than in HLA-DQ2.2(+) cells. Tyrosine present in the gliadin fragment establishing a bond to HLA-DQ2.5(+) APCs may be responsible for binding stability [44]. In this way, it facilitates antigen presentation and may explain the more frequent occurrence of HLA-DQ2.5 in patients with celiac disease than other antigens involved in the presentation of gliadin fragments. For this reason, HLA-DQ2.5 antigens are more frequently detected in patients with celiac disease than HLA-DQ2.2; however, the presence of HLA-DQ2.2 alone does not unequivocally exclude celiac disease. When conducting genetic tests, potential laboratory errors and misinterpretation of results should be taken into account [45].

## 5. Summary

As a result of the deepening knowledge on the etiopathogenesis and course of celiac disease, enormous progress has been made in recent years, which has directly translated into modifications of the criteria for diagnosing celiac disease. Today we know that some patients who met the diagnostic criteria for celiac disease years ago may not develop celiac disease, for instance, due to a lack of appropriate histocompatibility antigens. At the same time, some studies indicate the imperfection of the techniques used to determine histocompatibility antigens. Furthermore, attention is drawn to other histocompatibility antigens not previously included in the diagnostic criteria, but which may also be important in the development of celiac disease. However, considering the frequent absence of HLA-DQ2 and -DQ8, re-evaluation of diagnoses made years ago seems necessary.

## 6. Conclusions

For patients with celiac disease diagnosed before 2010, based on the 2020 ESPGHAN criteria, it is advisable to verify the previous diagnosis considering genetic criteria.

## Data Availability

The original contributions presented in the study are included in the article, further inquiries can be directed to the corresponding author.
